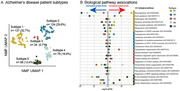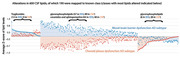# Alzheimer’s disease subtypes based on cerebrospinal fluid proteomics have distinct CSF lipid profiles

**DOI:** 10.1002/alz.083975

**Published:** 2025-01-09

**Authors:** Betty M. Tijms

**Affiliations:** ^1^ Alzheimer Center Amsterdam, Amsterdam UMC, Amsterdam, Netherlands; Alzheimer Center, Department of Neurology, Amsterdam university medical center, Amsterdam Netherlands

## Abstract

**Background:**

Disease mechanisms underlying Alzheimer’s disease (AD) are heterogenous amongst patients. We recently identified five distinct AD subtypes in cerebrospinal fluid (CSF) proteomic data with data‐driven techniques (Figure 1). Two of these subtypes were characterised by brain barrier dysfunction: one with choroid plexus dysfunction, and another with blood‐brain barrier dysfunction. Since most lipid transport takes place across these barriers, we compared these two subtypes on CSF lipid levels.

**Method:**

We included 419 individuals with abnormal amyloid across the clinical spectrum (i.e., AD) and 196 controls with intact cognition and normal AD biomarkers from the Amsterdam Dementia Cohort and related studies with CSF proteomic subtyping available. Next, we performed untargeted complex lipidomics with CSH‐QTOF mass spectrometry. Of 3532 lipids detected, 270 could be mapped to known classes. We compared AD barrier subtypes to controls on CSF lipid levels, controlling for sex and age with general linear models.

**Result:**

Of the 3532 lipids compared to controls, blood‐brain barrier dysfunction AD subtype had mostly increased levels of 302 lipids (148 with a known class; Figure 2, all p<0.05), whereas the choroid plexus subtype had mostly decreased levels of 314 lipids (163 known class;, all p<0.05) with an overlap of 150 lipids. These lipids included mostly glycerophospholipids, ceramides, and sphingomyelins. The blood‐brain barrier AD subtype further had increased levels of 14 tryglicerides, which were unaltered in the choroid plexus subtype. No specific alterations in CSF levels of known lipid classes were observed for the choroid plexus subtype.

**Conclusion:**

We previously identified 5 AD subtypes with distinct underlying mechanisms, including two subtypes with involvement of either the blood‐brain barrier or the choroid plexus. The subtypes with different type of brain barrier dysfunction had opposite alterations in CSF levels of lipids from specific classes. This implies that these barriers have a distinct role in lipid metabolism and transport alterations in AD, which may require specific treatments.